# Axonal projections of Renshaw cells in the thoracic spinal cord

**DOI:** 10.1002/phy2.161

**Published:** 2013-11-24

**Authors:** Shane A Saywell, Timothy W Ford, Peter A Kirkwood

**Affiliations:** 1 School of Health Sciences, Queen's Medical Centre, University of NottinghamNottingham, NG7 2HA, U.K.; 2 Sobell Department for Motor Neuroscience and Movement Disorders, UCL Institute of NeurologyQueen Square, London, WC1N 3BG, U.K.

**Keywords:** Axonal projections, interneurons, recurrent inhibition, Renshaw cell, respiratory neurons, thoracic spinal cord

## Abstract

Renshaw cells are widely distributed in all segments of the spinal cord, but detailed morphological studies of these cells and their axonal branching patterns have only been made for lumbosacral segments. For these, a characteristic distribution of terminals was reported, including extensive collateralization within 1–2 mm of the soma, but then more restricted collaterals given off at intervals from the funicular axon. Previous authors have suggested that the projections close to the soma serve inhibition of motoneurons (known to be greatest for the motor nuclei providing the Renshaw cell excitation) but that the distant projections serve mainly the inhibition of other neurons. However, in thoracic segments, inhibition of motoneurons is known to occur over two to three segments (20–40 mm) from the presumed somatic locations of the Renshaw cells. Here, we report the first detailed morphological study of Renshaw cell axons outside the lumbosacral segments, which investigated whether this different distribution of motoneuron inhibition is reflected in a different pattern of Renshaw cell terminations. Four Renshaw cells in T7 or T8 segments were intracellularly labeled with neurobiotin in anesthetized cats and their axons traced for distances ≥6 mm from the somata. The only morphological difference detected within this distance in comparison with Renshaw cells in the lumbosacral cord was a minimal taper in the funicular axons, where in the lumbosacral cord this is pronounced. Patterns of termination were virtually identical to those in the lumbosacral segments, so we conclude that these patterns are unrelated to the pattern of motoneuronal inhibition.

## Introduction

The Renshaw cell has been an object of fascination for spinal cord investigators since 1941, when their defining action, recurrent inhibition of motoneurons, was first demonstrated in the lumbosacral cord (Renshaw 1941, 1946; Eccles et al. 1954). Recurrent inhibition was initially thought to be absent from phrenic (Gill and Kuno 1963) and thoracic (Sears 1964b) motoneurons. Subsequently, however, it has been demonstrated to be a widespread phenomenon, present in the cervical (Lipski et al. 1985; Hilaire et al. 1986; Brink and Suzuki 1987), thoracic (Kirkwood et al. 1981), and sacral segments (Jankowska et al. 1978). The strength of recurrent inhibition has been shown to be greatest at the same segmental level as the motoneurons activating the inhibition (Eccles et al. 1961; Hamm et al. 1987), decreasing in strength with increasing distance.

More recently, Renshaw cells have been assigned an important role in defining the sequence of events in the development of cellular identity and of circuits in the spinal cord (Alvarez et al. 2013), even though the functional role of these iconic cells remains elusive (Alvarez and Fyffe 2007). Various cellular and circuit properties may help identify this role, including details of Renshaw cell morphology and projections, which so far have been only partially described. Intracellular labeling has shown the following. Renshaw cells in the lumbar cord are located toward the tip of the ventral horn (Jankowska and Lindström 1971; Van Keulen 1979; Lagerbäck and Kellerth 1985b; Fyffe 1990) and have a multipolar or fusiform morphology (Fyffe 1990), with few sparsely branched dendrites that project for relatively short distances. Their axons have been followed in the ventral funiculus up to 3 mm from the soma, with dense collaterals being given off close to the soma and the extent of the collateralization then decreasing with distance from the soma (Van Keulen 1979; Lagerbäck and Kellerth 1985a). The dense collateralization within the region of the motor nuclei closest to the soma is consistent with the strongest inhibition being evident in those motor nuclei. Nevertheless, the full extent of Renshaw cell projections has been demonstrated to be considerably wider than this, including, in the lumbar cord, up to 12 mm from the soma, as shown by antidromic activation (Jankowska and Smith 1973). The functional role of the more distant projections is uncertain. Jankowska and Smith (1973) hypothesized that the dense proximal projections of Renshaw cells provided inhibition of motoneurons, whereas the more distant ones served the other known connections of Renshaw cells, that is, inhibition of other Renshaw cells, of Ia interneurons or of ventral spinocerebellar tract neurons.

However, in thoracic segments, not only have the axonal projections been demonstrated to be even wider (at least two segments, up to 40 mm, on either side of the segment containing the Renshaw cells) but also these more distant projections have been shown to subserve motoneuronal inhibition (Kirkwood et al. 1981). Here, we report new observations made during experiments directed to intracellular recording and labeling of thoracic interneurons in general (Saywell et al. 2011). We took the opportunity to include in this study a few Renshaw cells from these segments, in the hope that we could shed more light on the organizational principles involved in the details of their axonal projections. Since the motoneuronal inhibition in these segments is much more rostrocaudally widespread than is the case in the lumbosacral cord, our hypothesis, following the views of Jankowska and Smith (1973), was that the axon collateralization would be much less focused to the region near to the Renshaw cell soma than it is for the lumbosacral segments.

A preliminary report has appeared (Saywell et al. 1998).

## Methods

### The preparation

Experiments were conducted according to UK legislation (Animals [Scientific Procedures] Act 1986). The data come from four cats of either sex, weighing 2.5–3.65 kg, all of them having also contributed to the data both of Saywell et al. (2007) and of Saywell et al. (2011). Animals were anesthetized with sodium pentobarbitone (initial dose 37.5 mg kg^−1^ i.p., then i.v. as required) and a bilateral vagotomy performed. Neuromuscular blockade was achieved by the use of gallamine triethiodide (subsequent to surgery, i.v., repeated doses 24 mg as required) and the animals were artificially ventilated via a tracheal cannula with oxygen-enriched air, to bring the end-tidal CO_2_ fraction initially to about 4%. A low stroke volume and a high pump rate (53 min^−1^) were employed so that events related to the central respiratory drive could be distinguished from those due to movement-related afferent input. A bilateral pneumothorax was performed and the end-expiratory pressure maintained at 2–3 cm H_2_O. CO_2_ was then added to the gas mixture to raise the end-tidal level sufficient to give a brisk respiratory discharge in the mid-thoracic intercostal nerves (typically 6–7%). During neuromuscular blockade, anesthesia was assessed by continuous observations of the patterns of the respiratory discharges and blood pressure together with responses, if any, of both of these to a noxious pinch of the forepaw. Only minimal, transient responses were allowed before supplements (5 mg kg^−1^) of pentobarbitone were administered. The animal was supported by vertebral clamps, a clamp on the iliac crest and a plate screwed to the skull. Rectal temperature was maintained between 37°C and 38°C by a thermostatically controlled heating blanket. Mean blood pressure, measured via a femoral arterial catheter, were above 80 mmHg throughout, maintained in a few animals by occasional i.v. infusions of 5% dextran in saline.

Three nerves were prepared for stimulation via platinum wire electrodes on the left side of one the segments to be used for intracellular recording (T7 or T8): (1) a bundle of dorsal ramus nerves (Kirkwood et al. 1988); (2) the external intercostal nerve; (3) the internal intercostal nerve (Sears 1964a). The left external intercostal nerve of T5 or T6 was prepared for recording efferent discharges, used to define inspiration. In three of the animals, the left dorsal roots of the segment used for intracellular recordings were cut during the experiment to allow subsequent specific identification of Renshaw cells.

A thoracic laminectomy was performed, the dura opened and small patches of pia removed from the dorsal columns of the segment(s) to be used for intracellular recording. Stimulating electrodes were inserted into the spinal cord of T10 segment (a pair of tungsten electrodes on each side, tips intended to be in the ventromedial and ventrolateral funiculi), as described by Kirkwood et al. (1988) and a shaped pressure plate lightly applied to the cord dorsum of the chosen segment, to aid mechanical stability. The laminectomy and nerves were submerged in a single paraffin oil pool constructed from skin flaps. At the end of the experiment, the animals were either killed with an overdose of anesthetic or perfused for histology (see below).

### Recording

Intracellular recordings were made from interneurons in the chosen segment by tracking in the left ventral horn with borosilicate glass microelectrodes filled with 2% Neurobiotin™ (Vector Laboratories Ltd, Peterborough, UK) and 0.5 mol/L K^+^ acetate in 0.01 mol/L Tris buffer (pH 7.4). Electrodes were introduced through the dorsal columns at an angle of 15° to the sagittal plane (Kirkwood et al. 1988) and with tracks 0.05 mm apart (microdrive: AB Transvertex, Stockholm, Sweden, step size 4 *μ*m). Electrodes were pulled on Model 753 electrode puller (Campden Instruments, Loughborough, UK) and bevelled to an impedance of 20–45 MΩ, (micropipette beveller BV-10; Sutter Instruments, Novato, CA). In order to ensure that the recordings were made mostly within the gray matter, tracks were located with respect to the positions of motoneurons as determined either by antidromic field potentials resulting from stimulation of the three nerves at 5× nerve threshold or by intracellular recordings from the motoneurons (cf. Kirkwood et al. 1988). When dorsal roots were intact, nerve stimulation was monitored from the cord dorsum by platinum wire electrodes mounted within the pressure plate, or after they were cut, from the peripheral stump of one of the roots, mounted on a platinum wire electrode. Renshaw cells (for identification, see Results) were sought at depths between around 2.5 mm from the surface and the ventral tip of the ventral horn (usually 3.0–3.5 mm deep, as indicated by the motoneuron positions).

Physiological data were recorded first. This included a short period of nerve stimulation and stimulation of the spinal cord, usually followed by a period with no stimulation, to allow assessment of the central respiratory drive and of the stability of the penetration. If the membrane potential was sufficiently stable, iontophoretic injection of neurobiotin was performed. Depolarizing current pulses (650 msec, 1 sec^−1^) of variable amplitude (typically 5 nA) were used. The response from nerve stimulation was set to occur a few ms before the start of each current pulse, so that the physiological state of injected neurons could be continuously monitored. Injection was terminated if the membrane potential declined to −20 mV, or sooner if the physiological response deteriorated too much. The current integrals for the four cells reported here were 26 nA min for one (B23J) and 72, 73, and 79 nA min for the other three. Membrane potentials were confirmed on exiting from the cell. Following termination of current injection, the electrode was withdrawn from the spinal cord and further penetrations restricted to positions at least 2 mm more rostral or caudal. The rostrocaudal positions of all injected cells were noted with respect to dorsal root entry positions or other surface marks.

All recordings were stored on magnetic tape. Data were acquired for computer analysis or display either online or offline (1401 A-D interface and Spike2 software; CED, Cambridge, UK). Both a low-gain d.c. version (amplification 50 × ) and a high-gain, high-pass filtered version (amplification 1000 ×, time constant, 50 msec) of the membrane potential were included.

### Histological procedures

Not more than four hours after the first injection of a cell (to allow for transport of label), the animal was heparinized and perfused through the left ventricle with phosphate-buffered saline (PBS), followed by 2 L of 4% paraformaldehyde in phosphate buffer (pH 7.4). Relevant segments of spinal cord were removed and, according to the hardness of the tissue, stored in either PBS or the same fixative overnight at 4°C.

The next day the pieces of spinal cord containing the injected Renshaw cell (two pieces 6 mm long for cell B18P, one piece 13–17 mm long for each of the other three cells) were cut either transversely (cell B18P) or parasagittally (the others) at 30 or 40 *μ*m on a vibrating cutter (Vibratome 1000; TPI, St Louis, MO). Sections were kept in serial order in multiple-welled arrays with nylon mesh bottoms and stored overnight in 0.1 mol/L PBS plus 0.3% Triton-X100 at 4°C. Alternate sections were kept as two separate series and one series reacted first. This series was incubated with either avidin-HRP (Sigma-Aldrich, Gillingham, UK) or ABC Elite (Vector Laboratories Ltd, Peterborough, UK) for 5–48 h. After six rinses in Tris buffer (pH 7.6), the sections were reacted with diaminobenzidine, nickel ammonium sulfate, and H_2_O_2_ in Tris buffer to reveal the labeled neuron and its processes. The progress of the reaction was followed by viewing the sections under a dissecting microscope. Once a well-stained neuron was identified or the background staining became prominent, the reaction was terminated by several washes in cold Tris buffer. Selected sections were mounted in glycerol and examined under the microscope to identify those containing part of the cell soma. The adjacent section, or part of it, containing the rest of the soma, was set aside for semithin sectioning and immunohistochemical processing (not reported here). The remaining sections were processed in an identical fashion to the first series, then all except the one section set aside were mounted on gelatinized slides, and air dried for at least 24 h. They were counterstained with neutral red (1% solution), serially dehydrated through acetone and cleared in xylene. The slides were then coated with DePeX mounting medium (BDH, VWR International, Lutterworth, UK) and coverslipped.

Sections were examined with an Axioskop microscope (Carl Zeiss UK, Cambridge, UK). Diameters of boutons or of axons were estimated to the nearest 0.5 *μ*m via an eyepiece micrometer and an oil-immersion objective (×100). In the parasagittal sections, the axons were only stained near the two faces of any section and measurements were made from all suitable axonal fragments. Particular care was taken when estimating diameters to avoid regions of the axon which appeared to be tapered as a result of fading toward the middle of the sections, or were of reduced width because the section was on a chord not a diameter. At the same time, apparent swellings resulting from kinking were avoided. In the transverse sections, measurements were made at rostrocaudal spacing of 0.3 mm and similar care was taken, the main hazard avoided here being overestimation resulting from kinking or from the flaring that can often occur at the face of the section. No correction for shrinkage was made for any of the dimensions quoted.

Graph plotting and the fitting of regression lines were performed with Origin software (OriginLab Corporation, Northampton, MA).

## Results

### Physiology

**Figure 1:**
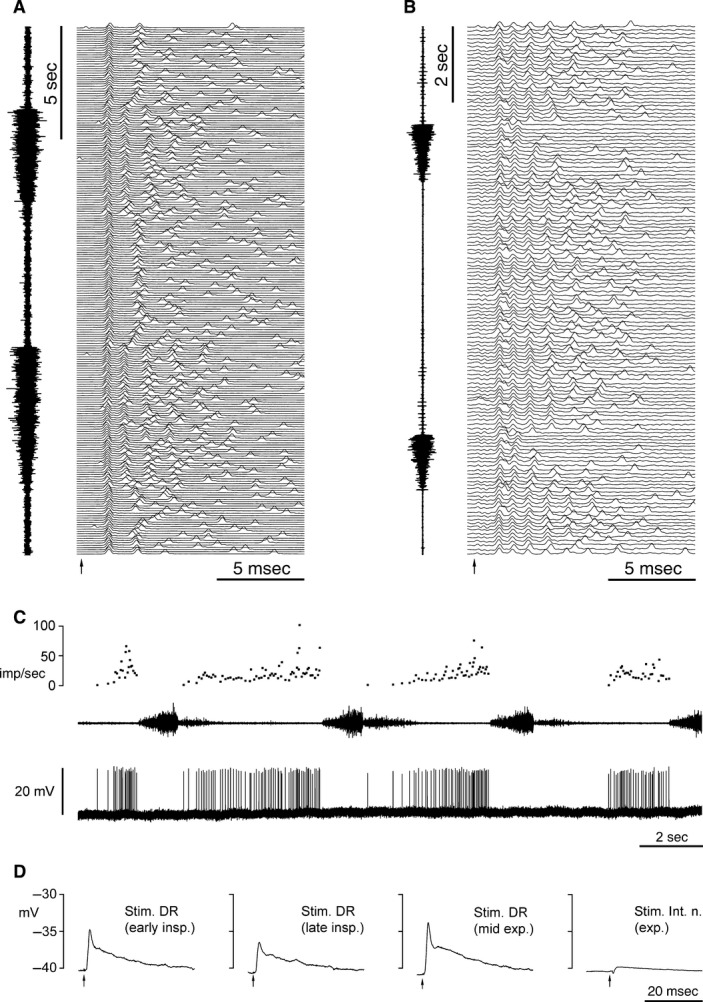
Electrophysiology of the Renshaw cells. (A, B) Respiratory variation in the responses to nerve stimulation (internal intercostal nerve for both, at the arrows). The intracellular recordings are shown in a raster format in time sequence from bottom to top. (A) Cell B23J, membrane potential −56 to −53 mV, spike amplitude 60–70 mV. (B) Cell B17P, membrane potential −26 mV, spike amplitude 10 mV. For each example, an external intercostal nerve discharge is shown at the right (T5 for A, T6 for B). (C) Spontaneous activity of cell B17P: intracellular recording shown in the bottom trace, firing rate (instantaneous frequency) on the top trace, T6 external nerve discharge in the middle trace. (D) Averaged intracellular responses from cell B18P to nerve stimulation, all within a single respiratory cycle, during early inspiration, late inspiration, and expiration, as indicated. First 3 panels, five sweeps averaged for each panel, stimulation to the dorsal ramus; fourth trace, 41 sweeps, stimulation to the internal intercostal nerve.

The properties of four Renshaw cells are reported here, one from each cat (Table 1). For three of these, the identification was unequivocal, via the presence of synaptically evoked responses to nerve stimulation in preparations with dorsal roots cut. The fourth cell, located deep in the ventral horn, was recorded with dorsal roots intact (B17P), but demonstrated identical responses to the others, viz a very high-frequency bust of discharges to nerve stimulation at a monosynaptic latency, typical of a Renshaw cell (Eccles et al. 1954) and recruited over a stimulus intensity range appropriate to motor axons, around 1.5–2.5× nerve threshold. It was therefore accepted as a Renshaw cell with almost the same degree of certainty as the others (Kirkwood et al. 1988; cf. Kirkwood et al. 1981). Three of the penetrations were axonal, as was clear from identifications of the recording sites, ≥1.3 mm from their somata and from the recording characteristics (spike shapes typical of axons, together with an absence of synaptic potentials, either spontaneous or evoked). The recording in the fourth cell (B18P) was either from the soma or from the axon close to the soma, as indicated by the presence of an excitatory postsynaptic potential (EPSP) (Fig. 1D).

**Table 1:** Properties of individual Renshaw cells

Cell identification	B17P	B18P	B22P	B23J
Dorsal roots	Intact	Cut	Cut	Cut
Segment	T8	T7	T7	T7
Spontaneous discharges	Expiratory	None	Expiratory	Inspiratory
Activating nerve	Internal	Dorsal ramus (+ internal)	Internal	Internal
Depth of recording, from dorsal surface (mm)	3.5	2.6	2.9	3.4
Membrane potential (best, mV)	−27	−40	−50	−55
Antidromic from T10	Yes	Yes	Not tested	Yes
Distance to T10 electrodes (mm)	29.5	27.5	–	36.5
Conduction velocity (m sec^−1^)	29.5	27.5	–	21.4
Sections	40 *μ*m parasagittal	30 *μ*m transverse	30 *μ*m parasagittal	30 *μ*m parasagittal
Dendritic spread (mm)				
Rostral	0.60	–	0.55	0.64
Caudal	0.60	–	0.69	0.40
Collateral spacing (mm)				
Rostral	0.65	0.41	1.2	1.4
Caudal	–	0.78	1.65	–
Mean axon diameter (*μ*m)				
Rostral	4.0	2.79	4.0	2.11
Caudal	3.25	2.98	2.58	2.39
Predicted conduction velocity (m sec^−1^) (caudal axon dia × 8, see text)	26.0	23.8	20.6	19.1

Three of the cells were spontaneously active, firing irregularly at relatively low frequencies (≤40 imp sec^−1^) and with respiratory modulation, expiratory for two (e.g., Fig. 1C) and inspiratory for the third (Table 1). These three cells responded to stimulation of the internal intercostal nerve with a burst of discharges that was modulated by the respiratory cycle in the same direction as their spontaneous discharges, that is, showing more spikes per stimulus and slightly shorter latencies (by about 0.1 msec) during the active phase (Fig. 1A and B). In the fourth cell (B18P), the spikes inactivated soon after penetration, leaving a long duration EPSP with a superimposed short-duration initial component, just as illustrated by Eccles et al. (1961). The amplitudes of both phases of the EPSP were modulated by respiration (Fig. 1D). Note that the absence of spontaneous discharges for this cell was not a consequence of the spike inactivation, as this absence was noted from spike discharges recorded extracellularly, immediately before penetration. Little respiratory drive potential was visible in this cell. When present, it was variable and no more than 1 mV in amplitude, with depolarization in inspiration.

A descending projection was identified by antidromic stimulation of the cord in all three of the cells where this was tested, involving a conduction from the stimulation site, 27.5–36.5 mm caudal. The latencies of the antidromic spikes (1.1–1.7 msec), with 0.1 msec allowed for utilization times, gave conduction velocities of 21.4–29.5 m sec^−1^ (Table 1).

### Anatomy

**Figure 2:**
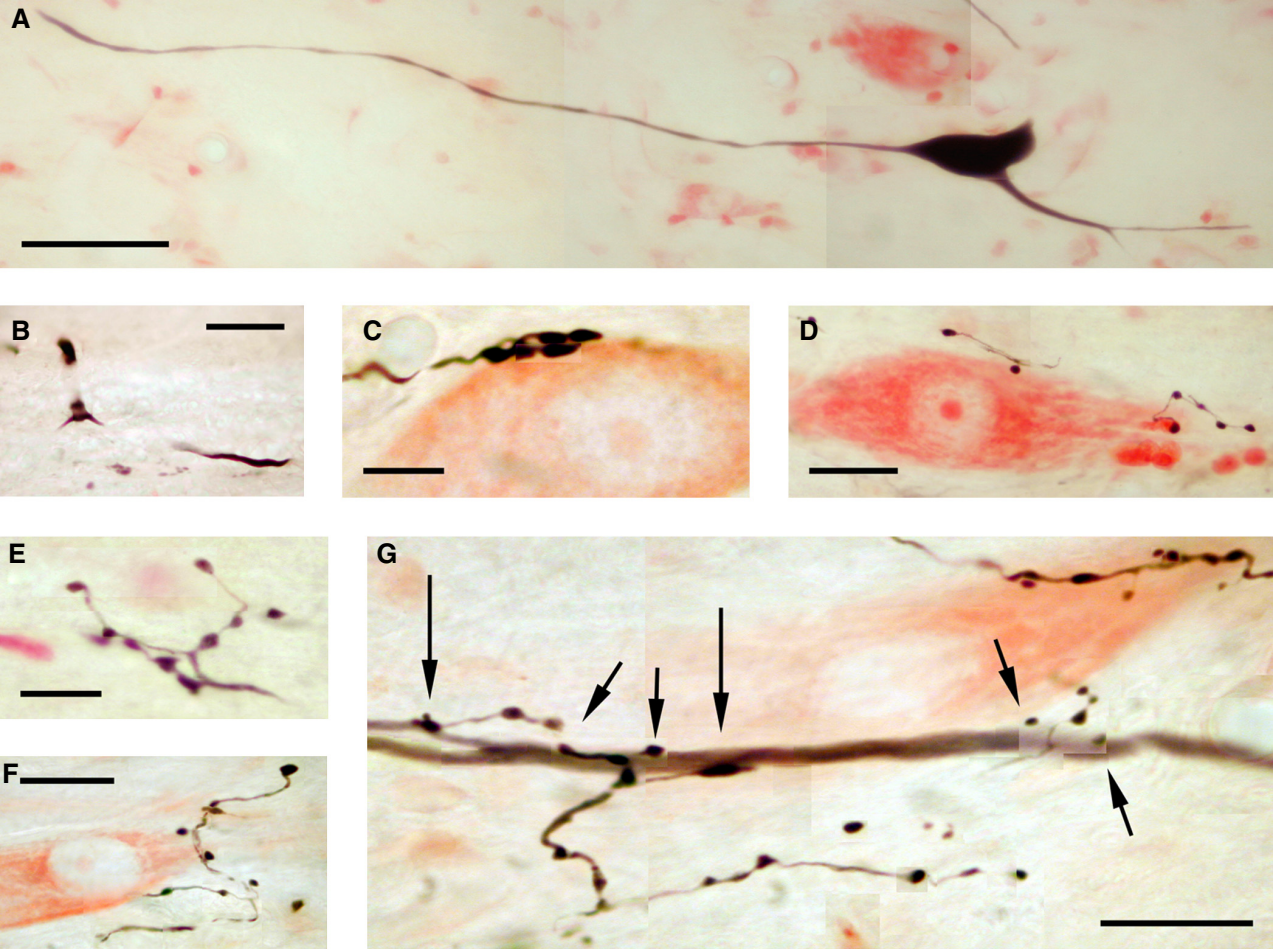
Morphological features of the Renshaw cells. (A) Soma and proximal dendrites (cell B23J). (B) Axonal branch point, just inside the white matter (cell B22P). (C) A cluster of five boutons, all closely apposed to a presumed motoneuron (B22P). (D) At least one bouton closely apposed to a presumed motoneuron (B23J). (E) Boutons from the most rostral collateral of cell B23J. (F) Boutons from the second most caudal collateral of cell B22P. (G) A series of boutons close to the dendrite of a motoneuron (antidromically identified from the internal intercostal nerve), also intracellularly labeled with neurobiotin. Boutons indicated by short arrows appeared to lie in different focal planes to the dendrite, but the two indicated by large arrows (one a very large bouton) lay in the same focal plane and no gap was visible. All panels consist of montages of several focal planes (A and D also two fields) and were adjusted for brightness and contrast in Photoshop (Adobe). For all panels, dorsal is at the top and rostral is to the left. Scale bars: A, 50 *μ*m; B, D, F, G, 20 *μ*m; C, E, 10 *μ*m.

**Figure 3:**
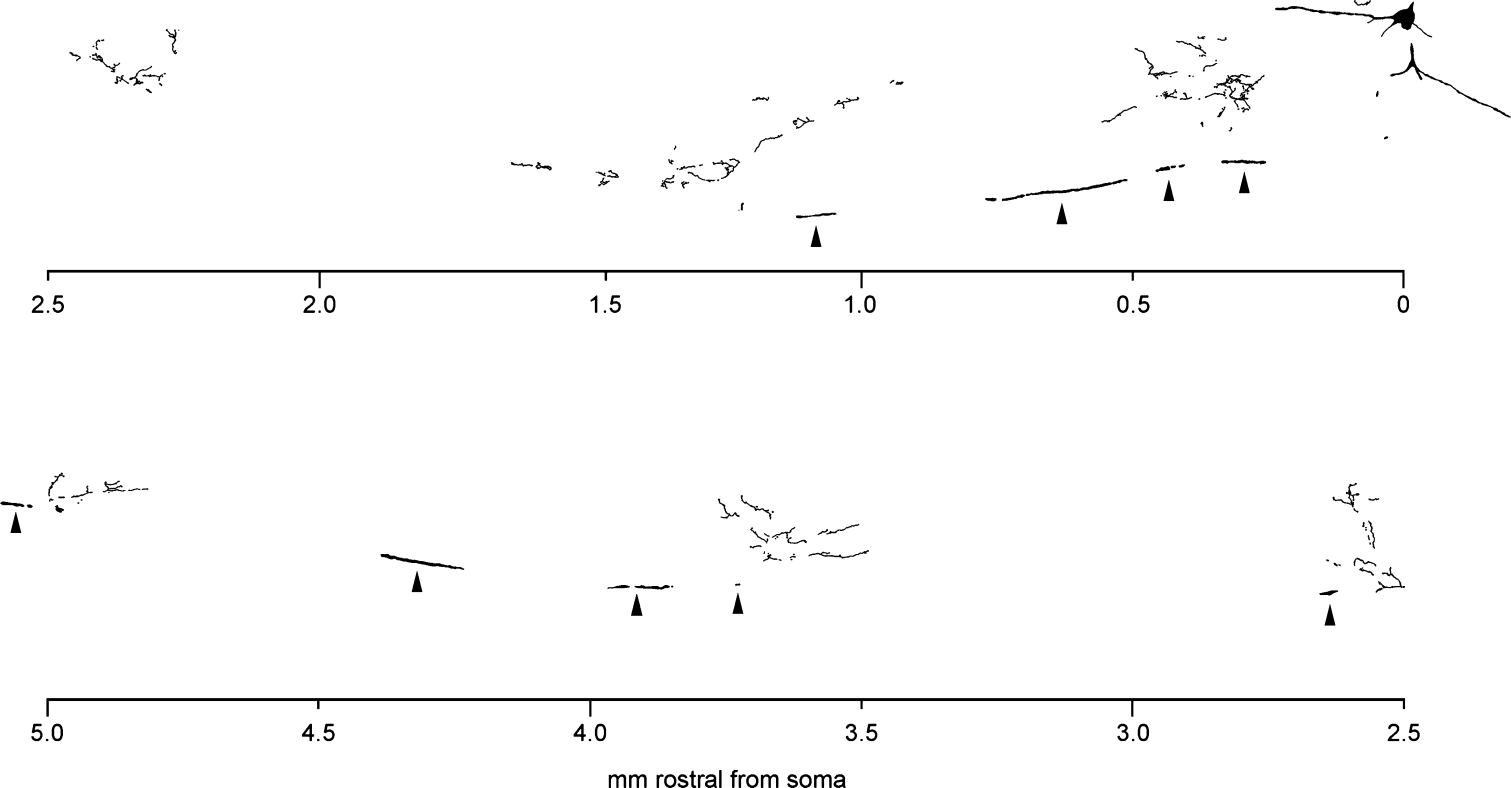
Drawing of all the labeled fragments in one parasagittal section, from cell B17P. The lower half of the figure should be read as continuous with the top half, aligned by the distance scale. The stem axon, in the white matter, is indicated by arrowheads. Drawing made via a drawing tube attached to the microscope. Dorsal is at the top of the figure, rostral is to the left.

The somata of three of the Renshaw cells were well labeled. The fourth soma, B18P, showed only granular reaction product and the dendrites were mostly not labeled. The somata were all located within 100–320 *μ*m from the ventral border of the ventral horn and 80–200 *μ*m from the lateral border. Only one half of each soma could be examined, the section containing the other half having been removed from the series. Nevertheless, it is clear that the somatodendritic morphology was generally very similar to that described for Renshaw cells in the lumbosacral cord (Lagerbäck and Kellerth 1985b; Fyffe 1990). Soma diameters were between 15 × 27 and 27 × 35 *μ*m. The cells possessed relatively few, tapered, generally slim, sparsely branched dendrites (Figs. 2A and 3). These projected within the ventral horn but hardly extended into the white matter, like the thoracic motoneurons of Lipski and Martin-Body (1987), but unlike most of the other thoracic interneurons described by Saywell et al. (2011). The dendrites were relatively short (as far as can be judged with one section missing in each instance), extending no more than about 0.7 mm rostral or caudal from the soma (Table 1). In this respect, they were similar to the other interneurons of Saywell et al. (2011) when soma size is taken into account. Dendrites were smooth; no spines were seen, although the distal dendrites of one cell were extensively beaded.

The axons of all four cells were well labeled. For all four, the axon branched at or close to the gray/white border (Fig. 2B) into a rostrally and a caudally running branch, located in the white matter, staying within 100 *μ*m of the ventral border of the ventral horn and extending to both ends of the spinal cord block examined. Axonal projections were exclusively ipsilateral. For all four axons, extensive collateralization occurred for the proximal region. As the axon progressed distally from the branch point, collaterals were given off at intervals of between 0.4 and 1.65 mm (Table 1). In some instances, collaterals were directly observed as they branched from the stem axon, but in other instances, their locations were deduced from the bouton distributions (see below). In any case, detailed reconstructions of collateral trajectories were not possible because of the missing section. Nevertheless, the general pattern of collateralization was very clear, sometimes even from a single section. Figure 3 shows a drawing of one such section, in this case the section adjacent to the missing one. Some parts of the main axon were located in adjacent sections, but also it was only stained near the two surfaces of the section, so it appears only intermittently in the drawing (arrowheads).

**Figure 4:**
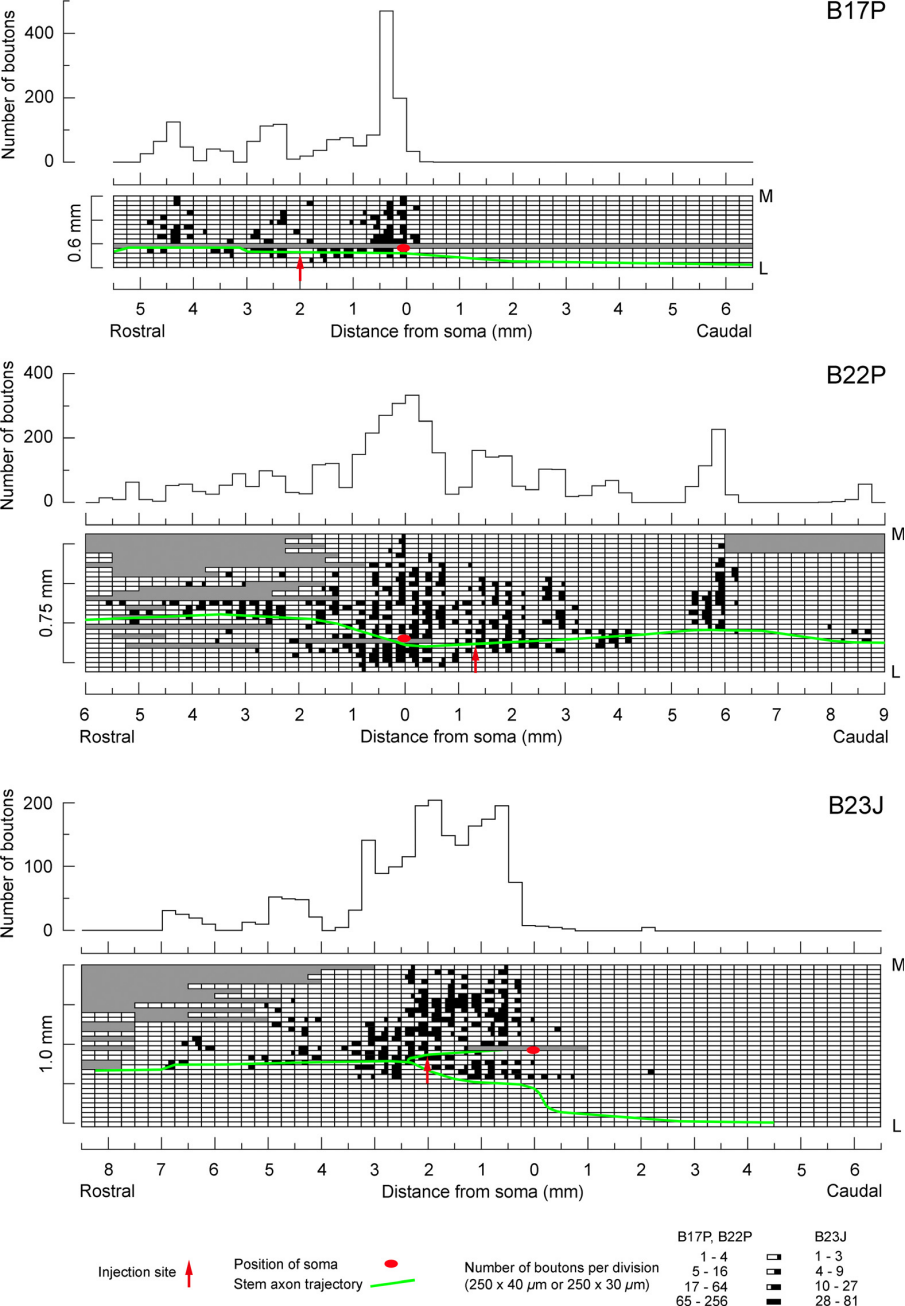
Bouton distributions for Renshaw cells. The three cells sectioned in the parasagittal plane are illustrated. For each cell, the upper plot shows the rostrocaudal distribution of boutons and the lower plot shows the bouton counts for individual sections, indicating also the mediolateral distribution (M, medial; L, lateral). Note the different section thickness for the first example compared to the other two and the nonlinear scales for the “number of boutons” in the lower plots. Positions of the soma, the stem axon and the injection site are as indicated. Note that the medial location of some of the boutons, particularly in B, means that these must also be located quite dorsally because of the dorsomedial orientation of the medial border of the ventral horn. Gray areas represent missing or unusable areas of individual sections.

Numerous boutons were observed, both *en passent* and *terminaux* (Fig. 2C–G). These were often larger (1–5 *μ*m in diameter) and more darkly stained than were most of those from the other thoracic interneurons labeled in the same series of animals (Saywell et al. 2011). Examples were readily found in close apposition to neutral red-stained somata (Fig. 2C and D). Although these represented only a minority of the boutons, as in Lagerbäck and Kellerth (1985a) or in Fyffe (1991), they presented an even clearer contrast with the other interneurons reported by Saywell et al. (2011), where such somatic boutons were exceptionally rare. For one cell, boutons were observed in close apposition to dendrites of a neurobiotin-labeled motoneuron, on third and fourth order dendritic branches at 320–400 *μ*m from the soma (Fig. 2G), similarly located to those reported by Fyffe (1991). Bouton distributions were measured for all of the axons, the three assessed in parasagittal sections being illustrated in Figure 4. For the proximal region, the boutons were distributed across a large part of the cross section of the ventral horn, including medial and lateral aspects, but as the collaterals became more distal from the soma, the number of boutons, and the areas they occupied became progressively smaller. It should be emphasized here that this was a real effect, not one of incomplete staining. All collateral branches in these three examples were seen to end with a bouton: none appeared to simply fade with distance and all but one were well stained. The one example was the most caudal collateral of cell B22P (at about 8 mm from the soma, Fig. 4), where both boutons and the collateral branches became progressively faint with distance from the stem axon, so this part of the plot is likely to be an underestimate. For axon B18P, collaterals originating more than 4 mm from the injection site (near the soma) also faded with distance. As examples of well-stained distally located boutons, Figure 2F is a photomicrograph from the next most caudal collateral of cell B22P, around 4.7 mm caudal of the injection site, and Figure 2E illustrates the most rostral collateral of cell B23J, around 5.0 mm from the injection site.

For all of the axons, the proximal, extensively collateralized region was spread over a restricted rostrocaudal distance (0.5 and 1.75 mm for the first two in Fig. 4 and 1.5 mm for B18P). For the fourth axon (B23J, third plot in Fig. 4), the plot was very similar, but the proximal peak of the rostrocaudal distribution was slightly wider (around 3 mm). However, within that region (not beyond), some of the boutons were rather faintly stained and may have been derived from other neurons or axons inadvertently penetrated (one or two weakly stained somata were also present). Thus, in this instance the extent of the peak may have been artifactually increased. For two of the axons (B22P and B18P), the more distal collaterals were seen both rostral and caudal to the soma, but for the other two, only rostral (Fig. 4 and Table 1). Note that this asymmetry was not related to the positions of the cells within the segment. All four of the somata were located within the most rostral 2.5 mm of the segment, as defined by the dorsal roots (T7 and T8 segments are about 10 mm long in the cat), that is, within about the most rostral 4 mm of the segment defined by the motoneuronal distribution (Meehan et al. 2004). Note also that the most rostral collaterals observed were located more than 4-mm rostral of the soma (Fig. 4) and therefore were located well within the next rostral segment.

**Figure 5:**
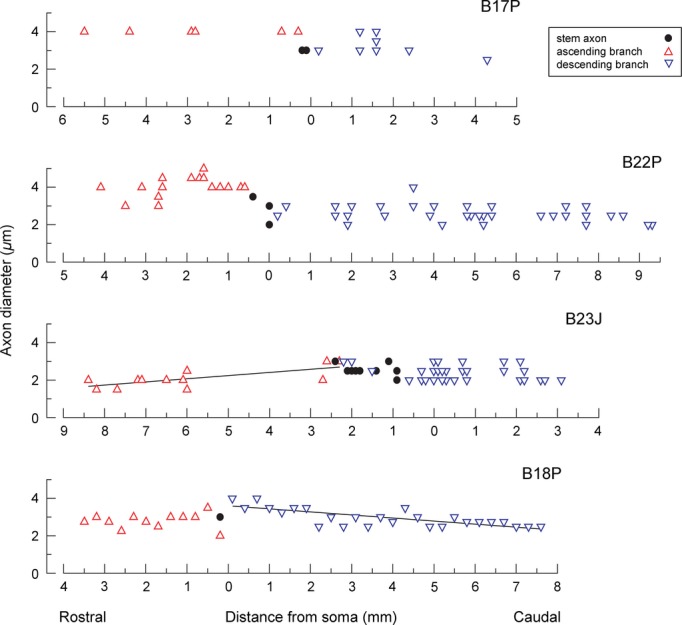
Axon diameters. The axon diameters for all four cells are shown plotted against the rostrocaudal distance from the soma. Regression lines are included for the two axon branches where the tapering was significant, as indicated by significant slopes of these lines. The symbols for the stem axon apply to measurements from the stem axon proximal to the main branch point.

The branch point was at about the same rostrocaudal location as the soma for three of the axons (within 0.2 mm), but was 2.4 mm rostral to the soma for the fourth (B23J). This might have been another reason why the central region of collateralization was more spread-out in this instance, as this region of collateralization largely corresponded to the rostrocaudal extent of the stem axon, proximal to the branch point. The diameters for each branch of the main axons in the ventral funiculus varied from 2 to 5 *μ*m (mean diameters in Table 1). Plots of diameter against rostrocaudal position are included in Figure 5 for all the axons. We investigated whether the axons might be tapered going either caudally or rostrally from the branch points by performing linear regression on the data for each of the eight axon branches. For only two of these (B23J rostral branch and B18P caudal branch) were the slopes significant (*P* = 0.0078, *P* < 0.001, respectively), and for both of these the slope was relatively modest (0.17 and 0.15 *μ*m mm^−1^, respectively). The mean diameters were larger for the rostral branch than the caudal branch for two of the cells and slightly smaller for the other two (Table 1).

## Discussion

The firing properties of the Renshaw cells in this study were similar to those previously reported for the thoracic spinal cord (Kirkwood et al. 1981, 1988; Hilaire et al. 1986). It is not possible to tell whether the respiratory modulation, either of the firing rate or of the responses to peripheral nerve stimulation, derives directly from a central input, or whether this is transmitted via motoneurons. The recordings were made under conditions of elevated CO_2_, where both inspiratory and expiratory motoneurons with axons in the internal intercostal nerve show brisk discharges in these segments (e.g., Fig. 1 of Kirkwood 1995). Motoneurons of the dorsal ramus nerves most likely would have been not only less active than these but also they would have shown a variety of respiratory patterns (Kirkwood et al. 1988; Saywell et al. 2007), so it is consistent that cell B18P, activated by the dorsal ramus nerve, was not firing spontaneously. The respiratory modulation of the EPSP in this cell is then of considerable interest. The cell showed very little respiratory drive potential, and what there was consisted of depolarization in inspiration, when the EPSP was smallest. This drive potential was therefore opposite in phase to that which might be expected to have provided shunting of the EPSP by inhibition, unless the inhibition was remote and/or coupled with simultaneous excitation, such as is the case for some thoracic motoneurons in the rat (de Almeida and Kirkwood 2010). Could this EPSP have been subject to presynaptic inhibition? Boutons that are themselves postsynaptic to other boutons have been observed on Renshaw cells (Lagerbäck and Ronnevi^1982^), but none of the individual boutons identified by Lagerbäck et al. (1981) as coming from motoneurons were of this category and, since then, other likely candidates for these boutons (synapses on Renshaw cells from primary afferents) have been identified, at least in the mouse (Mentis et al. 2006).

In general, we do not know what proportion of the respiratory modulation of the thoracic Renshaw cells arises by phasic excitation and how much by inhibition. A contribution from phasic excitation is certain, from the motoneurons known to be active with respiration. But then Renshaw cells are known to receive substantial inhibitory inputs, as shown physiologically (Wilson et al. 1964; Ryall 1970) and anatomically (Alvarez et al. 1997). However, the extent to which that inhibition is phasic with respiration (e.g., from other Renshaw cells) remains still unclear. In the measurements of Kirkwood et al. (1981), there were, at the most, only hints of recurrent excitation, which may be caused by inhibition between Renshaw cells (Ryall 1970).

An important new observation was the antidromic activation of the Renshaw cell axons from T10, two to three segments (27.5–36.5 mm) more caudal. This confirms directly what was deduced indirectly by Kirkwood et al. (1981) from the distribution of intersegmental recurrent inhibition, that the axons of the Renshaw cells in the thoracic cord extend 2–3 times further than do those of the lumbosacral cord described by Jankowska and Smith (1973). The conduction velocities measured here are consistent with the latency measurements of recurrent inhibition from Kirkwood et al. (1981) and are generally close to the values from the lumbosacral cord reported by Jankowska and Smith (1973).

The axon diameters measured here, being mostly constant with distance, are different from the axon illustrated by Lagerbäck and Kellerth (1985a), which showed marked tapering (by about 70%) over 3 mm. The axonal diameters here can also be compared directly with their measured conduction velocities. Predicted values of conduction velocities were calculated from mean values of diameter measured from the caudal branches of each individual axon, divided by a *g* value of 0.75 (Hildebrand and Hahn 1978) (to predict total fiber diameter including myelin), and multiplied by a factor of 6 (Hursh 1939) (i.e., multiplied by a total of 8). These values (Table 1, last line) were remarkably close to the measured values and suggest not only that there is an absence of tapering in the early parts of the funicular axons but also that, contrary to the results of Jankowska and Smith (1973), this lack of tapering may continue for the whole distance used for the measured conduction velocities (27.5–36.5 mm).

All four axons here projected both rostrally and caudally, so individual axons must be presumed to participate in the recurrent inhibition of both the adjacent segments (Kirkwood et al. 1981). Thus, there was no evidence of specialization of individual axons to project only rostrally or caudally. Given this, the absence of collaterals from the caudal branch for two of the axons is intriguing. One of these axons was injected rostral to the branch point, so for this one it might be thought that restricted transport of the label past this point might be an explanation. However, the axon in the caudal region appeared to be well stained and there were no indications of even weakly stained collaterals or boutons. The second axon was injected in the stem axon just caudal to the branch point, and caudal branch of the axon appeared as well stained as the rostral branch. This asymmetry of the collateral distribution is therefore unlikely to be artefactual and remains a feature requiring further investigation. In fact, asymmetry with regard to this branch point is not unique to intercostal segments: 3/5 of the cells reported by Lagerbäck and Kellerth (1985a) were without a caudal axonal branch, and Alvarez et al. (2013) noted that the caudal branch of the axon appeared later in development than the rostral branch. Finally, note the differences in diameter in the rostral and caudal branches for the axons illustrated in Figure 4.

A further question that arises is whether the extent of the spread of individual collaterals would continue to decline with distance going further into the adjacent segments and beyond. We suggest that this is unlikely because the strength of the inhibition in adjacent segments can be as high as 80% of that in the segment stimulated (Kirkwood et al. 1981), which suggests that there would actually need to be an enlargement in the collateral spread somewhere in the adjacent segment. Perhaps this might occur in the rostral part of the segment, where the concentration of motoneurons is greatest (Meehan et al. 2004).

In fact, the general axonal morphology observed here, including the branched stem axons and the general arrangement of the collaterals and their spacings, was remarkably similar to that shown by Van Keulen (1979) and Lagerbäck and Kellerth (1985a) in the lumbosacral cord (also see Fyffe 1991). The extent of the staining here allowed measurements to be made over rather longer rostrocaudal distances than in those previous studies, perhaps on account of using neurobiotin instead of horseradish peroxidase, or perhaps because of the intraaxonal site of injection. However, the important result is that this similarity of axonal morphology was seen despite the extended nature of the Renshaw cells’ projections in the thoracic cord, and despite the extended distribution of recurrent inhibition reported by Kirkwood et al. (1981). The only difference that could be related to this extended projection was the minimal taper in the funicular axons in this study. Thus, the overall pattern of collateralization, with dense projections close to the Renshaw cell soma, is unlikely to be related specifically to the recurrent inhibition of motoneurons, which is well known in the lumbosacral cord to occur predominantly close to the Renshaw somatic locations. This pattern must then represent some other functional or developmental property.
